# Immunopotentiating properties of chimeric OprF-OprI-PopB protein against* Pseudomonas aeruginosa* PAO1 in the infected burned rat model

**DOI:** 10.22038/IJBMS.2022.61448.13595

**Published:** 2022-03

**Authors:** Fattaneh Sabzehali, Hossein Goudarzi, Mehdi Goudarzi, Alireza Salimi Chirani, Mohammad Hossein Yoosefi Izad

**Affiliations:** 1 Department of Microbiology, School of Medicine, Shahid Beheshti University of Medical Sciences, Tehran, Iran

**Keywords:** Cloning, Expression, Granulocyte-macrophage- colony-stimulating factor, Immunity, Vaccine

## Abstract

**Objective(s):**

*Pseudomonas aeruginosa*, as an opportunistic pathogen, is known to cause nosocomial infections among patients suffering from burn injuries and also cystic fibrosis patients. The objective of our research was to develop a novel vaccine against *P. aeruginosa*.

**Materials and Methods:**

A recombinant *P. aeruginosa* subunit vaccine based on the outer membrane proteins, including the OprF-OprI region and its major protein in the type III secretion system, PopB (called FIB protein) was formulated. To induce a robust immune response, our preferred regions were conjugated to a carrier protein, GMCSF (Granulocyte-macrophage colony-stimulating factor). FIB protein’s immunogenicity with and without adjuvant was evaluated in vaccinated rats and also burned rat models, which were subcutaneously challenged by the PAO1 strain of *P. aeruginosa*.

**Results:**

Antibody levels were increased in sera of rats in this study. Assessment of the resident memory CD4+ T cells in splenocytes from vaccinated rats demonstrated that the FIB conjugated with GMCSF could cause higher responses in comparison with other groups. Moreover, immunization with the FIB plus adjuvant protein could improve interferon-gamma (IFN-γ) production, interleukin 17A (IL-17A), and IL-4, contributing to elicit humoral and cellular immune responses and decreased post-infection bacterial loads after PA challenge, pathology, and inflammatory cell infiltration.

**Conclusion:**

These observations demonstrated that FIB conjugated with GMCSF can be a putative vaccine candidate against *P. aeruginosa* in burnt rat models.

## Introduction

Increasing emergence of bacterial infections caused by *Pseudomonas* *aeruginosa *strains, which are intrinsically resistant to a wide range of antibiotics, has become one of the biggest challenges in clinical therapy, which can remarkably lead to increased mortality and morbidity mainly in immunocompromised patients (1). *P. aeruginosa* is a significant cause of burn wound infections by several different virulence factors such as alginate, outer membrane proteins, ExoU/S/A, proteases, rhamnolipids, secretion systems, hemolysin, phenazine, and pyocyanin. *P. aeruginosa* can form biofilm and also delay or prevent wound healing, which can lead to fatality. Since the multi- or extensively drug-resistant (MDR, XDR) strains have been reported as a major global public health threat for the at-risk patients, vaccination, immune- or phage-therapy (according to evolving translational strategies) are the promising approaches to combat this pathogen (2). Over preceding decades, *P. aeruginosa* vaccine development attempts have been focused on virulence factors interfering in *P. aeruginosa* pathogenesis, including flagellum, lipopolysaccharide (LPS), pili, alginate, outer-membrane proteins (OMPs), and exotoxins (3). Due to lack of proper animal models, serotype variation of LPS and flagella, and difficulties in executing clinical trials in high-risk populations, no proper vaccine against *P. aeruginosa* has been confirmed for human application (4). Among these antigens, the use of OMPs, including OprF and OprI, have been determined, as vaccine antigens are extremely encouraging (5, 6). Besides, type III secretion systems (T3SS) such as PopB can be considered as proper vaccine candidates. OprF/I, which is surface-exposed, is one of the most conserved components in different *P. aeruginosa* strains with varying LPS serotypes. OprF/I can be recognized by the immune system causing opsonophagocytic antibody production and can also be considered a potential vaccine candidate. PopB is a soluble and highly conserved component of T3SS, which can lead to pore formation in the upper part of the conduit to transfer effector proteins, including ExoT, ExoY, ExoS, or ExoU, into target cells and cause cell death (7). In the absence of opsonophagocytic antibodies, PopB can evoke strong Th17 responses and make antibody-independent protection from lethal *P. aeruginosa* pneumonia (8, 9). Since Th17 plays a vital role in initiation and adaptation of host antibacterial defenses, PopB, as a vaccine candidate, can be a proper choice to provoke Th17 and other immune particles. In this way, to make antibody-independent and antibody-dependent protection, we produced a chimeric antigen including a collection of peptides based on the outer-membrane (OprF/I) and T3SS (PopB) regions. This chimeric peptide was accompanied by the carrier molecule called GMCSF to enhance immunogenicity (10). GMCSF is a main immune modulator and growth factor, which responds to various stimulations and is recognized as a potent inducer of Th17 cytokine. Currently, several studies have revealed that GMCSF can be secreted from Th17 cells in murine experimental autoimmune encephalomyelitis models (11, 12). Since *P. aeruginosa* is the major pathogen in burned hosts, administering the FIB vaccine subcutaneously is vital for protection. By vaccination and challenge studies in burned rat models, we assessed the vaccine’s immunogenicity, protective potential, and bacterial burden reduction. It was determined that subcutaneous vaccination, with FIB protein and GMCSF as a potent adjuvant, stimulates both humoral and cellular immune responses in burned models.

## Materials and Methods

***Bioinformatics analysis to design the recombinant FIB protein***

The chimeric FIB protein was designed by multi-step stages: (i) The sequences of OprF, OprI, and PopB in *P. aeruginosa* were retrieved from the UniProt database at http://www.uniprot.org (P13794, P11221, and Q9I324) and aligned using ClustalW (13). (ii) The “Gly-Gly-Gly-Gly-Ser-Ser-Ser” sequence was determined as an integrating linker to join OprF to OprI and OprI to PopB. (iii) The Physicochemical and immunogenic properties were determined using the ProtParam tool (http://web.expasy.org/protparam/) (14) and SOLpro (15). (iv) The secondary structure was predicted as α-helices, β-sheets, and coil structures using the MINNOU server (http://minnou.cchmc.org). Moreover, covariances of residues were analyzed using CoeViz (16) and PSIPRED (17).Furthermore, the weak polar interactions among the side-chain of aromatic rings and amide hydrogen backbone(Ar-HN interactions) were predicted via the IMTECH server (18). (v) The three-dimensional (3D) structure of FIB protein, which was not determined, was predicted using the Modeller v9.19 software package. Ten top models were selected based on their highest DOPE score, refined using GalaxyRefine (http://galaxy.seoklab.org/cgi-bin/submit.cgi?type=REFINE) (19), and validated via PROCHECK software (20), (Vi) the 3D hydrophilicity and hydrophobicity modeling of the FIB protein were predicted by the ProtScale software (https://web.expasy.org/protscale/) and visualized using the PYMOL program (21). (Vii) The Linear B cell epitopes were determined using the BepiPred algorithm (22). The chimeric FIB sequence between NdeI and XhoI restriction enzyme sites was optimized, synthesized, and cloned into the pET-22b (+) cloning vector by Biomatik Company (Ontario, Canada) with >85% w/w purity and validated using PCR and sequencing.

***Bacterial strains***

The *P. aeruginosa* PAO1 “ATCC 7853” strain from the Iranian Pasteur Institute was utilized and cultivated in a shaking Luria-Bertani (LB) medium (Merck Co, Darmstadt, Germany-250 rpm) at 37 °C.

***Confirmation, expression, and purification of His-tagged FIB protein***

The Plasmid pET-22b (+) accompanied with FIB were transformed into *Escherichia coli *(*E. coli*) DH5a and *E. coli* BL21 (DE3) pLysS (Novagen, USA) host bacteria to reproduce and express, respectively. The chimeric plasmid transformation into DH5a cells was performed using the heat shock method, in the presence of Cacl2. After transformation, colony PCR and sequencing were performed to confirm gene cloning. The recombinant plasmid was extracted via DNA-spin ^TM^ Plasmid DNA kit (Roche kit Plasmid DNA extraction, USA) (23) and boiling. Colony-PCR was utilized to confirm the presence of the synthetic gene in the pET-22b (+) plasmid vector (24). Colony PCR was accomplished using T_7_ terminator and T_7_ promoter primers under the following program: denaturation at 95 °C for 5 min followed by 29 cycles at 95 °C for 45 sec, 58 °C for 30 sec, and 72 °C for 30 sec, and one min-final extension at 72 °C. PCR products were analyzed utilizing 1% agarose gel electrophoresis. Later, the cloned protein into *E. coli* BL21 (DE3) pLysS was grown at 37 °C in LB medium, supplemented with ampicillin (Amp; 100 μg ml^-1^), and the FIB expression was induced in the presence of 1 mM isopropyl-beta-thio galacto pyranoside (IPTG, Sigma, St. Louis, MO, USA) for 4, 6, and 12 hr. A colony containing pET-22b (+) without *oprF-oprI-popB* gene was applied as a negative control. Before disrupting via cell suspension in lysis buffer (containing: 20 mM Tris-HCl, 0.5 M NaCl, 1 mM PMSF, 10 mM Imidazole, 0.3 %Triton X, pH=8.0) and sonication (20 cycles of 30 sec, 4 °C) (UP50H, Germany), the induced cells were harvested at 4 °C and 4000 rpm, 15 min by centrifugation. Then, the cell lysate, after centrifugation (4000 rpm, 10 min) was analyzed by sodium dodecyl sulfate-polyacrylamide gel electrophoresis (SDS–PAGE) using a 12.5% polyacrylamide gel, visualized via standard Coomassie Brilliant Blue G-250 for 18 hr and destained with 45% methanol 10% glacial acetic acid solution. For purification of His-tagged fusion protein, transformed cell harboring pET-22b (+)- *oprF/I*, and *popB* plasmid was grown at a large scale in LB broth. When the optical density (OD) reached 600 nm, approximately 0.5 (T_0_), 1 mM IPTG was added and incubated with shaking at 37 °C for 12 hr. Then, the cell fractions were separated by centrifugation at 7000 rpm and 4 °C for 10 min. The pellet was washed with phosphate-buffered saline (PBS), re-suspended in 4 ml lysis buffer, sonicated as described before, and centrifuged at 8000 rpm for 10 min. The pellet got mixed with Ni-NTA resin to purify fusion proteins, according to the manufacturer’s manual. The chromatography column was washed with different buffers, including binding buffer (containing Urea 8M) to lysis proteins, denaturing washing buffer C, renaturation washing buffers to decrease the volume of urea, native washing buffer (containing 20 mM imidazole) to expel irreverent proteins (25). Finally, recombinant protein was eluted using elution buffer (containing 250 mM imidazole) (Ni-NTA Agarose; Qiagen). The purified protein was dialyzed in formulation buffer (1.9 mM Na_2_HPO_4_. 2H_2_O, 8 mM NaH_2_PO_4_, 0.15 M NaCl) and PBS containing 0.1 M sucrose, 0.04% Tween 20, pH = 7.2, at 4 °C overnight to remove imidazole. The purity of fractions was analyzed by 12.5% SDS-PAGE and Bradford protein assay (26). Moreover, western blot analysis was performed using vertical electrophoresis in SDS-polyacrylamide gel in a 12.5% gradient to separate, confirm, and evaluate antigenicity characteristics and immunogenic properties of the FIB protein. Briefly, the FIB protein was transferred to the Immobilon-PSQ PVDF Membrane (Merck, Germany) at 80 V overnight. The blot was blocked (5% skim milk in TBST containing 20 mM Tris–HCl, 100 mM NaCl, and 0.1% Tween 20, pH 7.6) at room temperature (RT) for 2 hr and washed three times. The membrane was incubated for 2 hr at RT with 6-His tag antibody horseradish peroxidase conjugate (Roche, Manheim, Germany) and diluted 1:2000 in Tris-NaCl, rewashed, incubated with the substrate (3, 3-diaminobenzidine containing 1% H_2_O_2_), and then washed with distilled water (27).

***Rat immunization***

In this experiment, groups of 6 to 7 week-old female Wistar rats were anesthetized via intraperitoneal injection (IP) of a mix of 0.3 ml (300 µl) of 10% ketamine and xylazine 0.1 ml (100 µl) (Ketanest, Pfizer Pharma GmBH, Berlin, Germany*)* in a sterile vial. All rats were kept in standard conditions (22 °C and 12/12 hr dark/light cycle). The experiments were performed under the Animal Ethical and Experimental Committee of the Shahid Beheshti University of Medical Sciences, Iran. Institutional and International Ethical Guidelines approved the protocols utilized. To experiment, the rats were immunized subcutaneously with FIB protein (50 µl; group 1), FIB protein accompanied by adjuvant (GMCSF was used as an adjuvant, 50 µl; group 2), adjuvant with Phosphate-buffered saline (PBS, 50 µl; group 3), and PBS (50 µl; group 4) on day 0 followed by a booster (25 µl) on days 14 and 28.

***Blood sampling***

Blood samples were collected from each rat’s retro-orbital venous sinus on days 0, 14, 28, and 42 before, and 44 post-challenge infections. The samples were left at RT for 15 min to clot the blood and then centrifuged for 5 min at 5000 rpm. Serum was obtained and stored at -20 °C.

***Immunization and measurement of rats’ antibody titer***

IgG2a, IgG2b, and IgG1, total IgG, IgM, and IgA (before and after challenge) titers in the rat serum collections were measured by the enzyme-linked immunosorbent assay (ELISA) in 96-well polystyrene microtiter plates (Greiner Labortechnik, Germany). Briefly, the standard microtiter plates were coated with a volume of 100 µl of a 1 µg/100 µl solution of the FIB protein in a 0.5 mol/l carbonate buffer, pH=9.6 overnight at 4 °C. After the coating stage, the plates were washed five times with 0.05 mol/l PBS and 0.05% Tween 20 (PBS-T) (Sigma-Aldrich) and blocked by 1% casein in PBS (1.5 mol/l) for 2 hr at 37 °C. The blocked plates were washed five times with PBS-T. 100 µl of the immune and control serum samples were diluted with 1:100 of 1% casein in PBS, added, and incubated at 37 °C for 2 hr. The plates were then incubated with anti-IgG, -IgM, -IgG1, -IgG2a, -IgG2b, and IgA HRP-conjugated goat anti-mouse antibodies (Southern Biotech Canada) at 1: 5,000 per well (100 µl) for 2 hr at RT and then for 1 hr at 37 °C. Plates were washed eight times with PBS-T and incubated with 100 µl of 3, 3′, 5, 5′-Tetramethylbenzidine (TMB; Sigma-Aldrich, USA) solution for approximately 10 min at RT. The enzymatic reaction was stopped by adding 100 μl of H_2_SO_4_ (2 mol/l), and absorbance at 450 nm was read using an ELISA plate reader (BioTek, Germany) (5).

***Burn challenge***

All burned female Wistar rats (weighing 150–280 g) were anesthetized with 100 μl of the mix of 10% ketamine-xylazine. The rats were placed over a sterile pad and fixed in a prone position. Eye ointment was used to prevent rats’ eyes from dryness through anesthesia. The dorsomedial side up to the middle of the rats’ neck was cleaned with alcohol, shaved, and burned with a heated cylindrical probe, which was made of iron alloy (1*1.5 inch) accompanied by 96% ethanol (0.5 ml) for 10 sec to create the third-grade burns. Rats immediately received 100 μl of acetaminophen (0.25 mg/ml) to prevent getting shocked and feeling the pain. Then, the rats were challenged with the suspension of PAO1 ATCC 7853 *P. aeruginosa* strain*. *This strain was inoculated in LB medium and incubated for 18 hr at 37 °C with shaking. According to Ian Alan Holder protocol (28), 250 CFU/ml bacteria from the fresh medium (1 ml) was centrifuged for 15 min at 5000 rpm. The supernatant fluid was discarded, and the pellet was washed twice with sterile PBS. The medium was then centrifuged as the last stage. The pellet was suspended in PBS (100 ml). On the 42^nd^ day, the burned area was dipped into 0.1 ml of *Pseudomonas*. The rats were placed into the special cages whose heights were more than the regular cages. Mortality and survival rates were recorded for 7 days, and also scars healing was observed and reported after 21 days.

***Cytokine flow cytometry***

Activation markers on stained splenic leukocyte populations were evaluated using BD Biosciences FACSCanto II Flow Cytometer (BD Biosciences, USA) and the data were analyzed using Flowjo software (version 10.4.2, USA). Briefly, spleens were extracted in aseptic condition and homogenized in RPMI-1640 supplemented with 200 μg/ml streptomycin, 10 mM Hepes, 200 IU/ml penicillin, 50 μM 2-mercaptoethanol (2ME), and 10% FBS (Sigma-Aldrich, Germany) to lyse red blood cells and prepare single-cell suspensions. Cells, which were 10^6^ per sample, were washed in PBS and incubated with the Trypan blue before antibody staining in order to separate dead cells. All cells were incubated with FcγR block (BD Bioscience) followed by surface antigen staining (anti-CD16/32, BD biosciences). Afterward, the following mAb were utilized for surface antigen staining, including Anti-mouse CD4 Fluorescein isothiocyanate (FITC)-conjugate, Anti-mouse CD3 Brilliant Violet 510 (BV510)-conjugate, Anti-mouse CD44 PerCP/Cyanine 5.5 (PerCP/Cy5.5)-conjugate, and Anti-mouse CD62L (PE)-conjugate (all from BD Biosciences). For intracellular staining of IFN-γ, IL-4, and IL-17, cells were counted and covered in 96-well plates with a complete Roswell Park Memorial Institute (RMPI)-1640 medium. 5 × 10^6 ^cells/ml spleen was simulated for 4 hr at 37 °C in 5% CO_2_ with PMA (10 ng/ml phorbol myristate acetate; Sigma) and ionomycin (1 μg/ml; Sigma), in the presence of Brefeldin A (10 μg/ml; Sigma). Then they were recovered and stained with trypan blue, incubated with FcγR block, fixed with 2% formaldehyde in PBS, and permeabilized with 0.05% saponin (Sigma) in PBS. Intracellular staining was performed with Anti-IFN-γ FITC-conjugate (BD Biosciences), Anti-IL-17A PE-conjugate (BioLegend), and Anti-mouse IL-4 PE/Cy7-conjugate (BD Biosciences) within only CD4^+ ^T cells to distinguish T-cell subtype. Fluorescence minus one (FMO) controls were stained in parallel. To define gates and evaluate non-specific staining, isotype controls were utilized. Data analysis was performed using BD FACSDiva Software and compensated in FlowJo (29).

***Opsonophagocytic killing (OPK) assay***

According to OPK assay, the sera titers decrease the number of live bacteria by more than half. Briefly, bacterial suspensions of PAO1 from *P. aeruginosa* were provided at an approximate concentration of 2×10^7^ CFUs in 1% bovine serum albumin (BSA) (30). Sterile sodium thioglycolate (0.5 ml; Sigma) was injected into the peritoneum to provoke macrophage. Four days later, the rats were killed with CO_2_ and immersed in 70% ethanol. Their abdominal cavities were opened and cold RPMI-1640 was injected (10 ml), afterward, they were aspirated and washed twice with sterile PBS. Rat macrophages were suspended at a final concentration of 2×10^7^ CFUs/ml in RPMI-1640 medium supplemented with 10% heat-inactivated fetal calf serum (FCS). A 3-week-old baby rabbit (Pasture Institute of Karaj, Iran) was bled and the developed serum (final concentration, 4%) was utilized as a complement source. To do this assay, the suspension of bacteria (2 × 10^7^ cells per well) was incubated with an equal volume of heat-inactivated specific polyclonal IgG of rat (at 56 ºC for 30 min) (concentration 1:10) at RT for 90 min and later washed twice with BSA (1% (w/v)) to eliminate excessive antibodies. Later, it was suspended in 200 µl of 1% BSA, 100 µl of rat macrophages was merged with 100 µl complement in a sterile 48-well microfuge plate (Greiner bio-one, Germany) and next incubated in a shaking incubator for 90 min at 37 °C. After (time 0) and 90 min of incubation at 37 ºC, 25 µl of the mixture was immediately removed, diluted in 225 µl saline, subsequently plated for bacterial enumeration, and cultured in MacConkey agar (Merck KGaA, Darmstadt, Germany). Control wells, which lacked serum, complement, or macrophage components were replaced by 100 µl of BSA. As the following formula, the percentage of killed bacteria in different groups was duplicated for each quantity and then it was calculated. The percentage of opsonophagocytosis = (1- (CFU of immune serum at 90 min / CFU of pre-immune serum at 90 min)) ×100. In general, ≥50 percent of the opsonic killing activity of immune serum was considered biologically significant.

***Histological analysis of burned skin***

Skin tissues were collected 10 hr post-infection from the burned rat models. Samples were fixed in 10% neutral buffered formalin, routinely processed, paraffin-embedded, and sectioned for hematoxylin and eosin (H/E) staining and evaluations. Afterward, each section was analyzed by an expert pathologist in terms of ulcerous area, granulated tissue, and neutrophil infiltration using optical microscopy at 100X and 200X magnifications (31).

***Statistical analysis***

All statistical analyses were executed using Prism version 8 (GraphPad Software Inc., USA). Comparisons between two or more parametric data groups were performed by ordinary one-way analysis of variance (ANOVA) followed by Tukey’s multiple comparisons test. Statistical analysis for evaluating nonparametric data was performed using the Kruskal-Wallis test with Dunns’ multiple comparison test. To urge correlation, the Pearson correlation coefficient test was used. One sample t-test was utilized to compare opsonophagocytic killing assays. The Log-rank (Mantel-Cox) test was applied to compare the survival distributions of samples. A *P*-value of <0.05 was regarded statistically significant.

## Results

***Sequence analysis and prediction of FIB protein***

**Figure 1:**
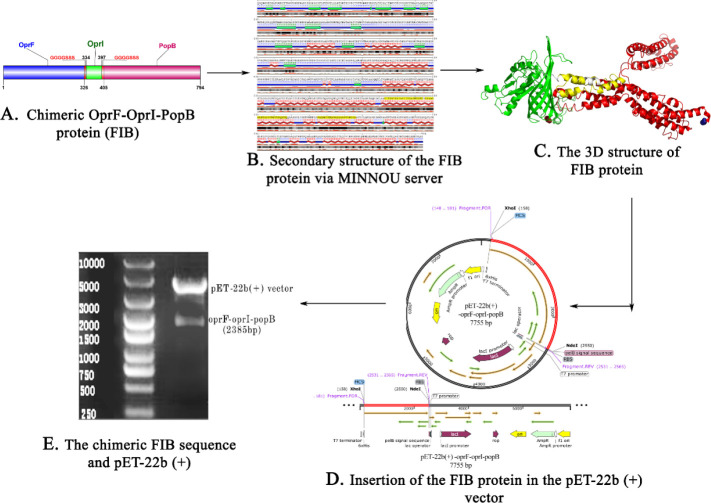
Vaccine design and synthesis of the FIB protein. (A) Schematic representation of *Pseudomonas aeruginosa* PAO1 antigenic construct consisting of the three regions of OprF1-326, OprI334-397, and PopB405-794. (B) The FIB protein secondary structure investigation via MINNOU server pointed that the construct was contained 70 β-sheet, 454 α-helix, and 270 coils. The blue line represented random coil while the β-sheet and α-helix structures presented with the green and red colors. The orange columns showed the confidence of prediction for each position. (C) OprF (green color), OprI (yellow color) PopB (red color) were located in the first, second, and third part of the 3D structure of FIB protein in which these sequences (OprF-OprI and OprI-PopB) via two flexible linkers (white color, [G4S3]) were attached. The N-terminal and C-terminal regions (red firebricks color and blue density) were displayed at the first and last of the sequence. (D) The chimeric fusion protein (2385 bp) accompany by its vector (pET-22b (+)-5493 bp) was synthesized and (E) digested with NdeI and XhoI restriction enzymes via BIOMATIK

The sequence alignment of OprF, OPrI, and PopB using ClustalW showed that all the sequences were conserved in different isolates of *P. aeruginosa* (Figures 1-S1, S2, and S3). The physicochemical characteristics and immunogenic features of this construct were theoretically predicted. PH was below 7 (acidic), instability index was below 40 (stable), the aliphatic index value was 82.09 (thermostable), negative grand average of hydropathy value was (hydrophilic), and this construct was extremely soluble (approximately 1.0). The antigenicity was (0.7756), immunogenicity was (0.70099), and it was non-allergenic (0). Moreover, the half-life of FIB fusion was about 0.8 hr and 10 hr in mammalian reticulocytes (*in vitro*) and *E. coli* (*in vivo*), respectively. The chimeric stability of the protein was confirmed. A schematic depiction of chimeric FIB domains is illustrated in Figure 1A. The second structure of the fusion protein was predicated and it was revealed that it mainly consisted of β-sheet and also α-helix at the endings. Due to the α-helix, the construct was extremely stable (Figure 1B). The eight weakly polar Ar (i)-HN (i+1) interactions in FIB protein were predicted via the NHPred server, in which the acceptor and donor residues and their respective positions were Phe^11^-Gly^12^, Tyr^15^-Phe^16^, Phe^16^-Thr^17^, Tyr^29^-Gly^30^, Tyr^35^-Phe^36^, Phe^36^-Leu^37^, Tyr^47^-Gly^48^, and Tyr^50^-His^51^. Due to lack of homology in the structure of some proteins, such as OprI, we had to develop through the HHPred, *ab intio* method, and the Modeller v9.19 software to predict the FIB protein structure (Figure 1C). The z-scores of the chimeric protein before and after structural refinement using the GalaxyRefine software were -5.08 and -6.22 correspondingly. The stereochemical quality, the overall quality factor, free energy folding, clash score, and maximum and minimum regions of hydropathicity associated with the chimeric protein were 71.07%, -587.50, 46.82, 3.044, and -2.989, respectively. These outcomes indicated that the FIB protein has a reliable structure. Furthermore, the sequence-based epitope prediction revealed that “LDAIYHFGTPGVGLRP,” “RGTYETGNKKVHGNLT,” “DVCSDSDNDGVCDNVD,” “SKVADLGGKFGSLAGQ,” “AKIGGKAAEMTASLAS,” “CSSHSKETEARLTA,” “ANRQADVQESRADLTT,” “ARAQARADEAYRKADE,” “VVRVQLDVKFDFDKSK,” “NINSDSQGRQ,” “NATAEGRAINRRVE,” “VCSDSDNDGVCDNVD,” “CSDSDNDGVCDNVDK,” “KAANRQADVQESRAD,” “AQKAQQTADEANERA,” and “EGHTDSVGTDAYNQK” would play the main role in provoking different parts of the immune system. In the next step, to enhance immunogenicity, the chimeric protein was conjugated with GMCSF as a potential carrier (Figure 1D). Double digestion of recombinant plasmid (pET-22b (+)- oprF/I and popB) led to two bands of 5493 bp and 2385 bp according to Linear pET-22b (+) and *oprF-oprI-popB* gene sizes, respectively. The enzymatic digestion is displayed in Figure 1E.

***Expression and purification of the FIB Protein***

**Figure 2:**
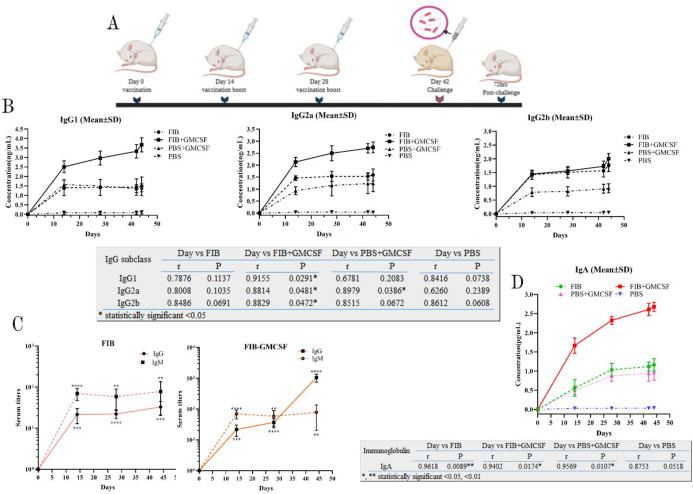
Laboratory study and in vivo antibody responses. (A) A schematic plan of the vaccination timeline. Rats were inoculated with FIB (G1), FIB/GMCSF conjugates (G2), PBS/GMCSF (G3), and PBS only (G4) and received a booster (25 µl) on day 14 and 28. On day 42, rats subcutaneously challenged with the *Pseudomonas aeruginosa *PAO1 strain. (B) ELISA titers. IgG1, IgG2a, and IgG2b anti-PAO1 antibodies in four groups of vaccinated rats (n = 8) were collected on the days of 14, 28, 42 (before infection), 44 (after infection) and due to Pearson correlation coefficient test revealed that the FIB protein's immunogenicity in FIB/GMCSF was higher than other groups. (C) IgM and total IgG anti-PAO1 antibodies, which were collected on the days of 14, 28 (Before infection), and 44 (After infection), represented that while the low anti-FIB IgM antibody titers could not be caused to isotype switching to IgG (Left picture), increasing anti-FIB/GMCSF could induce a significant IgG antibody response (Right picture). Since two PBS/GMCSF and PBS groups could not induce antibody titers a lot before and after challenging, we considered performing ELISA for the FIB and FIB/GMCSF. (D) IgA anti-PAO1 antibodies collected on the days of 14, 28, 42 (Before infection), and 44 (After infection) represented that according to Pearson correlation coefficient test, FIB/GMCSF protein conjugates could induce mucous immunity into other groups. The comparisons implemented using Tukey's multiple comparisons test. Asterisk denotes statistically significant differences (* for *P*≤ 0.05, ** for *P*≤ 0.01, *** for *P*≤ 0.001 and **** for *P*≤ 0.0001). R reveals the Pearson correlation coefficient. Error bars indicate mean ± SEM

PCR amplification from a part of the* oprF-OprI-popB* gene of *P. aeruginosa* ATCC 7853 genomic DNA led to 635 bp bands (Figure 2-SA). The presence of oprF-oprI-popB containing sequence in the plasmid pET-22b (+) was approved by the DNA sequencing method. After expression, the His-tagged chimeric protein was purified by Ni-NTA resin and identified by SDS-PAGE and western blotting (Figures 2-SB and 2-SC). The protein concentration in the pellet (insoluble state) was 83.2 kDa. 

***Antibody production in a rat model of vaccination***

To enhance the immunogenicity of the FIB protein, the vaccine candidate was formulated with GMCSF. FIB, FIB+GMCSF, GMCSF+PBS, and PBS, which were subcutaneously administered to 6-7-week-old female rats (Figure 2A). On days 14, 28, 42 (before infection), and 44 (after infection), the subtypes of IgG, including IgG1, IgG2a, and IgG2b, were evaluated using ELISA. The results indicated that anti-FIB protein-specific IgG1, IgG2a, and IgG2b were increased in the immunized rats by the FIB+GMCSF protein compared with unconjugated FIB and other groups (*P*<0.0001) (Figure 2B). Due to the class switching to IgG could be carried out following the increase in the production of anti- *P. aeruginosa* IgM, in the FIB+GMCSF vaccinated rat, a significant output of anti-FIB protein-specific IgM antibodies compared with unconjugated FIB were obtained in pre-and post-infection (Figure 2C). IgA plays a crucial role in mucous membrane immune function to combat microbial infections. We observed that the amount of IgA produced in the FIB+GMCSF group was higher than in other groups before and after infection (Figure 2D). 

***Cell*** ***-mediated*** ***immunity reaction triggered*** ***by the*** ***FIB vaccine*** ***before and after infection***

**Figure 3:**
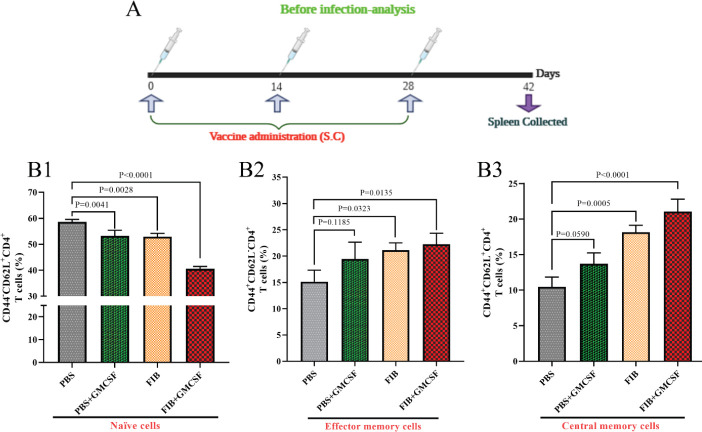
The investigation of TCD4+ production on day 42 (pre-challenge). (A) Due to the schedule, rats have immunized with the FIB chimeric recombinant vaccine, and then splenic cells obtained on day 42. (B) The naïve (CD44-CD62L+; B1), effector memory (CD44+CD62L-; B2) and central memory (CD44+CD62L+; B3) proportions of splenic CD4+T cells. Group comparisons analyzed by ANOVA followed by a Tukey's multiple comparison test for the CD44CD62LCD4 cell population. Numbers in the plots indicate the frequency of cells in each region (mean±SD) in the presence of appropriate controls including isotype and FMO controls

On day 42 (Figure 3A), the proportion of CD4^+^T cells from 3 chosen rats of each group, including CD44^-^CD62^+^ and other control groups (PBS and PBS+GMCSF), was higher than the vaccinated groups; there was not any significant difference between FIB and PBS containing GMCSF (Figure 3B1). The proportion of CD44^+^CD62L^+^ and CD44^+^CD62L^-^ phenotypes was considerably increased in FIB+GMCSF vaccinated rats’ splenic cells compared with other groups. These results illustrated central memory and effector CD4^+^T cell stimulation, which had a prominent role in encountering microbial pathogen (Figure 3). Moreover, the lymphocytes gating strategy and the contour plots used to distinguish naïve, effector, and memory cells by flow cytometry are displayed in Figure 3-S.

**Figure 4:**
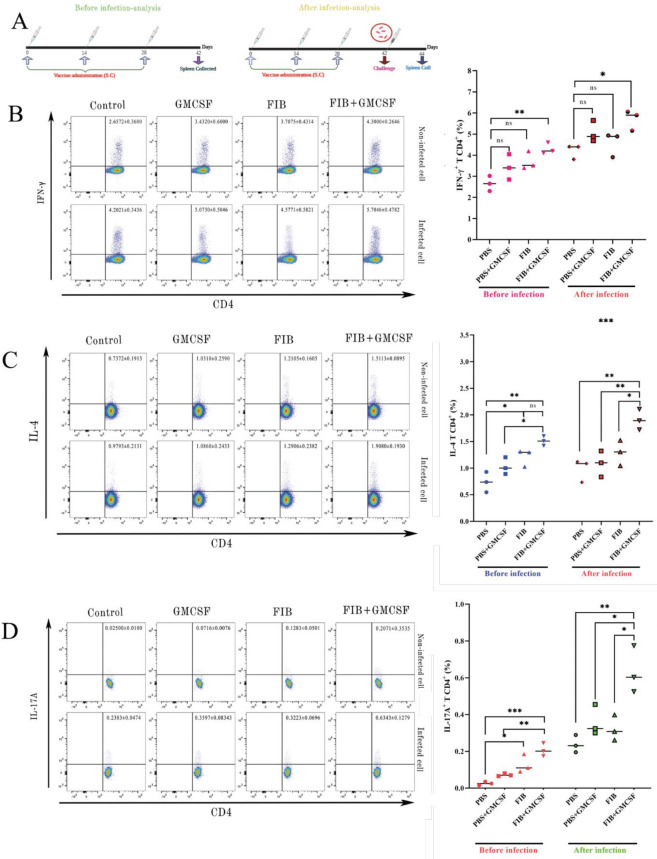
Developed generation of IFN-γ, IL-4, and IL-17A by CD4+T cells of vaccinated rats. (A) According to the schedule's vaccine, rats have immunized with the FIB chimeric recombinant vaccine, and splenic cells removed on day 42. On that day, the rats infected with P. aeruginosa, then, 72 h later, their spleens accidentally obtained to analyze the cytokine's expression of CD4+T cells. Representative dot plots and bubble charts exhibiting the frequencies of splenic CD4+T cells producing IFN-γ (B), IL-4 (C), and IL-17A (D) increased before and after infection of P. aeruginosa. Numbers in the plots indicate the frequency of cells in each region (mean±SD) in the presence of appropriate controls including isotype and FMO controls. The asterisks refer to statistical significance: ns= not significant, *P≤ 0.05, **P≤ 0.01, ***P≤ 0.001

On days 42 and 44 (before and after *P. aeruginosa *challenge*; *Figure 4A), the proportions of CD4^+^T cells producing IFN-γ, IL-4, and IL-17A in spleen cells were defined via flow cytometry technique to clarify the effector function of these cells provoked by the FIB vaccination. A similar gating strategy like the one in Figure 3-S was performed. Various cellular populations, including IFN-γ, IL-4, and IL-17A, exhibited via dot plots and bubble charts were illustrated in Figures 4B, 4C, and 4D. As exhibited in these figures, both in infected and non-infected rats, the frequency of splenic CD4^+^T cells representing IFN-γ, IL-4, and IL-17A were increased in FIB+GMCSF compared with other groups. These results proposed that protection presented by the FIB vaccine was reliant on these T cell populations.

***Evaluation of opsonic killing activity***

**Figure 5:**
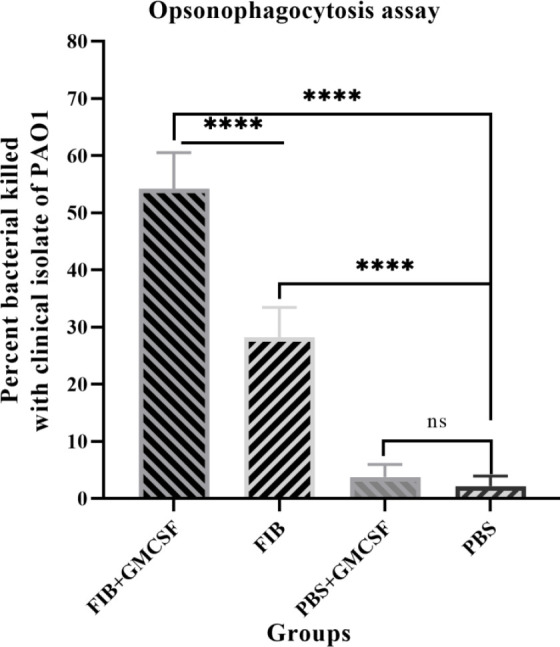
Comparison of the opsonic killing activity of serum rats against *Pseudomonas* *aeruginosa* strain PAO1. In this part of the examination, the strain of PAO1 was incubated with the suspension of bacteria (2 × 10^7^ cells per well) were set with an equal volume of heat-inactivated specific polyclonal IgG of rat (concentration 1:10) in the presence of rabbit complement. The significant opsonic killing activity recognized when a specific FIB+GMCSF antiserum treated with PAO1. No cross-reactivity identified between normal rabbit serum and the strain of PAO1. Bars represent the means of triplicate determinations, and the error bar intimates SD. The asterisks express the groups, which are significantly different (*P*≤ 0.0001), and ns mean not significant

OPK activity in serum was evaluated by incubating *P. aeruginosa* PAO1 strain with the anti-FIB /or anti-FIB+GMCSF/or anti-PBS+GMSF/or anti-PBS antibodies and rat macrophages in the presence of rabbit complement to find out the bioactivity of the FIB protein IgG *in vitro* condition. As displayed in Figure 5, FIB+GMCSF antisera at 1: 10 dilution significantly increased the OPK of *P. aeruginosa* strain PAO1 (52.02%) compared with the serum isolated from the control group rat (*P*≤0.0001). This increase of phagocytosis compared with other immune groups was probably due to the anti-recombinant IgG FIB + GMCSF specificity. Although the opsonic killing activity of FIB only antisera against the PAO1 strain was significantly higher than that of serum from control rats, it was not considered biologically significant (≤50%).

***Protection against the lethal strain of P. aeruginosa***

**Figure 6:**
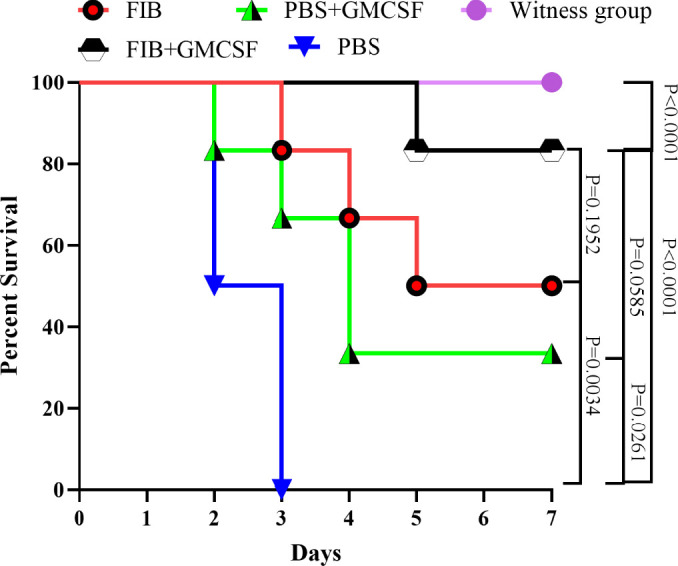
Assessment of mortality of *Pseudomonas* aeruginosa strain PAO1 between immune and non-immune groups of rats during 7 days. Any PBS received rats (control group; n=6) had perished through the 3 days. The vaccinated FIB protein's survival rate conjugated GMCSF challenged with *Pseudomonas aeruginosa* strain PAO1 was more potent than other groups

After the challenge with *P. aeruginosa* strain PAO1 on day 42, the total body weight was evaluated at 24 hr post-challenge (Figure 4-S) and the rat mortality was screened for 7 consecutive days. All PBS received rats (control group) had died during the 3 days after the burn challenge (Table 1-S). Compared with control groups, the survival rate of rats vaccinated with the FIB protein conjugated with GMCSF challenged with *P. aeruginosa* strain PAO1 83.3% were determined based on the Log-rank test (Mantel-Cox) test (*P*<0.0001). Not only we observed a significant difference between FIB + GMCSF and the other groups, but also we found a significant difference between FIB only and PBS groups (*P*=0.0034) (Figure 6). Overall, along with the adjuvant, the FIB vaccine candidate could be immunogenic in the rat host.

***Decreasing inflammation via FIB vaccination***

**Figure 7:**
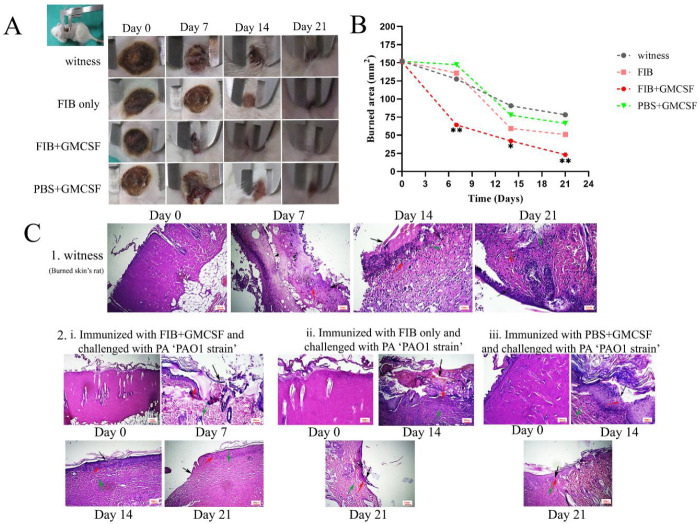
Healing of deep second-degree burn of rat skin with FIB vaccine candidate. (A) Images of deep second burn wounds at 0, 7, 14, and 21 days. (B) Burn area of rats’ skin lesions. Time points are the averages for measurements of different wounds. *P≤0.05 and **P≤0.01 compare the FIB, FIB+GMCSF, and PBS+GMCSF groups with the witness group. These experiments carried out twice with almost similar results. (C) Hematoxylin-eosin staining of skins from immunized rat and witness 10 hours after infection, and on days 7, 14, and 21. Representative histopathological sections from 5 rats group of FIB+GMCSF, 3 rats group of FIB, 2 rats group PBS+GMCSF and 6 rats group witness are shown (Red arrow: Neutrophils, Green arrow: Granulation tissue, Black arrow: Ulcer area, Magnification = 100X, 200X)

We stained histological sections with H/E to assess tissue burn injury and the resulting immune response to the burn challenge and infection 10 hr after infection (day 0) and also on days 7, 14, and 21. Regarding the histological analysis, FIB chimeric vaccine immunized groups presented reduced epithelial disruption, inflammatory cell infiltration, vascular leakage, bleeding, bacterial infection, and tissue damage compared with other groups including the witness group (Figure 7-C1, 7-C2ii). The results illustrate that the severity of skin damage was remarkably lower in the FIB+GMCSF group than in FIB and displayed the protective effect of FIB vaccination due to decreased bacterial burden and pathology. It showed that the burned areas significantly healed in the FIB+GMCSF group compared with the other groups (Figure 7B).

## Discussion

The increasing antibiotic resistance of *P. aeruginosa* confers a need for vaccines, however, due to its extensive diversity, there have been obstacles to developing effective vaccine candidates (28). It has been revealed that despite secretion of IFN-γ and IL-17 from CD3-activated thymocytes, higher additional activation of T-cell receptor against *P. aeruginosa* resulted in higher frequencies of IFN-γ but not IL-17 in the splenic cells. Therefore, increasing IL-17, CD4, and CD8 T cells can improve powerful immune response initiation and recruitment of immune cells such as neutrophils to the infection site (32). Based on our findings, PopB as a translocon of T3SS and other bacterial proteins such as FliC activate the cytosolic sensor NLR family CARD domain-containing protein 4 inflammasome, resulting in pyroptosis and leading to lung injury and impaired *P. aeruginosa* clearance via reducing interleukin 17 and antimicrobial peptide production (33). Hence, utilizing the antigen-specific antisera against PopB can influence immune cells and trigger IL-17 secretion of T cells (34). IL-17 increases inducible lung epithelial antimicrobial peptide expression, leading directly to enhanced bacterial clearance. Therefore, it has enough potential to be a proper vaccine candidate that can stimulate immune cells. Moreover, we found that OMPs, including OprF and OPrI, are responsible for activating the immune responses against *P. aeruginosa* and increased clearance of this pathogen (35, 36). The mucoid strains of PA were highly resistant to complement mediate lysis compared with non-mucoid strains due to the attendance of a long O-Side-Chain-Lipopolysaccharide, which intervenes with deposition of C3b and C9 on bacterial surface. Therefore, utilizing the antigen-specific antisera against OprF and OprI causes a significantly enhanced phagocytosis via increasing antibody titer against mucoid and non-mucoid *P. aeruginosa* strains (37). Since Gly4 Ser3 linker has a small size and endures in aqueous solvents due to its hydrogen bonds forming with water molecules, it was selected as a linker to create trivalent recombinant protein (38). To enhance immunogenicity, we conjugated the FIB protein with GMCSF carrier protein, which synergizes with IL-17 in inciting granulopoiesis, Dendritic cells (DCs) expansion, and functional enhancement of DCs, which can induce both cellular and humoral immune responses and provide protection against PA in burned rats. As multiple sequence alignment investigations were done in this study, it was suggested that the utilized OprF/I and PopB were more conserved within the pathogenic strains of *P. aeruginosa*, including PAO1, B136-33, LESB58, M18, NCGM2, PA7, PA14, DK2, SCV20265, and Pak (Figures 1-S1, -S2, and -S3). The physicochemical and immunological results showed the chimeric FIB protein was stable, soluble, immunogenic, non-allergenic, and contained the proper secondary and three-dimension structures, which could provide suitable immune responses (Figures 1 A–C). Based on former studies, immunization with OprF/I could improve opsonophagocytosis and PopB elicited Th17 responses via recruitment of neutrophils (39, 40); hence, we decided to combine them to make a stable immunity. In this study, the synthesis of FIB protein was performed via the BIOMATIK Company. Then, we confirmed, expressed, and purified the FIB protein in *E. coli *BL21 (DE3) pLysS, which could produce a T7 lysosome to make the target genes more stable. According to prior studies, inducing neutralizing antibodies and promoting bacterial clearance have been introduced as the main immunotherapy strategy and protection against *P. aeruginosa* infection (41). Therefore, in the current study, in the absence or presence of GMCSF, vaccination with FIB protein led to IgM, total IgG, IgG1, IgG2a, IgG2b, and IgA in sera of burned rats (Figures 2 B–D). Furthermore, it was indicated that the presence of IgGs-mediated opsonophagocytosis in the sera of burned rats resulted in significant bacterial clearance in *in vitro* conditions (Figure 5). We understood the proportion of total IgG and IgA increased against FIB+GMCSF in vaccinated and challenged groups, which resulted in effective humoral and mucosal immunity against *P. aeruginosa*. Moreover, the rate of IgG1, IgG2a, IgG2b titers, IgG1 and IgG2a especially increased in vaccinated rats, which indicates balanced Th1-Th2 responses. These findings are compatible with Schaefers and Laghaei’s studies which have determined that such antigens could provoke balanced Th1/Th2 immune responses (40, 42). In addition, some scientists indicated that immunization with a combination of OprF, OprI, and flagellins could produce high-affinity IgG antibodies and reduce the load of bacterial burden in their selected animal hosts (39, 43). To determine the role of tissue-resident memory (TRM) cells in the splenic cells of vaccinated rats, we conducted a flow cytometry experiment to recognize the percentage of TRM cells (n=3 for T_N_, T_CM_, and T_EM_).  The results revealed that immunization with the FIB+GMCSF vaccine could decrease TN cell percentage and increase the proportion of T_CM_ and T_EM_ cells compared with the other groups (Figures 3B1–B3). In the current study, we implied that TRM has a reasonable role in response to subcutaneous vaccination. Numerous investigations have revealed a host protective role of IFN-γ, IL-4, and IL-17A to activate B cells and recruit immune cells in *P. aeruginosa* infection (8, 44). Our results illustrated that the FIB+GMCSF vaccine triggered CD4^+^T cells to produce IFN-γ, IL-4, and IL-17A compared with other groups before and after challenging *P. aeruginosa*, which could respond immediately against PA stimuli without an additional priming phase (Figures 4B–D). Sen-Kilic *et al*. 2019 report was consistent with our results and demonstrated that the intranasal peptide-based FpvA-KLH conjugate vaccine with curdlan caused an increase in the recruitment of CD11b^+^ DCs and TRM cells in mice with *P. aeruginosa* pneumonia (29). An additional study demonstrated that the PcrVNH protein could activate cellular and humoral immunity via increases in the levels of IgG1, IgG2a, and IgG2b antibodies, and IL-4, IL-17, and IL-1β cytokines (45). In the present study, since FIB antigen increased the survival rate in rats infected by strain PAO1 of *P. aeruginosa*, this can be proof of its efficacy (Figure 6). We acknowledged that our protective efficacy data were consistent with the Yang study (2017), which designed a trivalent vaccine (Pcr-OprI-Hcp) conjugated with Al(OH)3 and evaluated its protective efficacy in pneumonia and burn mouse models. He urged immunization with this vaccine candidate could stimulate strong immune responses and decrease the bacterial loads (46). In addition, a lower bacterial injury accompanied by inflammatory neutrophil infiltration and high protective efficacy was observed in the current study, which may be defined by adequate DCs and Th1/2 immune determinants to the skin tissue. Consistent with animal and preclinical studies (47), the presence of GMCSF as a potent adjuvant accelerated wound healing by expression of some important cytokines. We implied that subcutaneous immunization accompanied by GMCSF could be an alternative layout for immunization against *P. aeruginosa*.

## Conclusion

Overall, these results offer evidence that the chimeric vaccine can increase the levels of antibodies and present protection against *P. aeruginosa* infection in burned rat models via increasing CD4^+^ tissue-resident memory T cells considering T_CM_ and T_EM_ cells, and evoking effective humoral and cellular immune responses, including induction of IFN-γ, IL-4, and IL-17A via ELISA and flow cytometry tests. The chimeric recombinant of the FIB vaccine has developed an opsonophagocytic killing procedure, decreasing the systemic pathogen distribution and inflammation in histological analysis. Therefore, this study illustrated that the FIB protein conjugate, GMCSF, was effective in rats and could restrict burned infections caused by *P. aeruginosa*.

## Authors’ Contributions

FS, MG, and HG prepared the draft manuscript; MG, FS, and ASCH Revised the paper; FS and MHYI Collected the data, designed the vaccine structure, performed experiments, evaluated the results, and analyzed the data.

## Conflicts of Interest

All authors declare that they have no competing interests. 
